# Timelines of translational science: From technology initiation to FDA approval

**DOI:** 10.1371/journal.pone.0177371

**Published:** 2017-05-08

**Authors:** Laura M. McNamee, Michael Jay Walsh, Fred D. Ledley

**Affiliations:** 1 Center for Integration of Science and Industry, Department of Natural and Applied Sciences, Bentley University, Waltham, Massachusetts, United States of America; 2 Department of Management, Bentley University, Waltham, Massachusetts, United States of America; Royal College of Surgeons in Ireland, IRELAND

## Abstract

While timelines for clinical development have been extensively studied, there is little data on the broader path from initiation of research on novel drug targets, to approval of drugs based on this research. We examined timelines of translational science for 138 drugs and biologicals approved by the FDA from 2010–2014 using an analytical model of technology maturation. Research on targets for 102 products exhibited a characteristic (S-curve) maturation pattern with exponential growth between statistically defined technology initiation and established points. The median initiation was 1974, with a median of 25 years to the established point, 28 years to first clinical trials, and 36 years to FDA approval. No products were approved before the established point, and development timelines were significantly longer when the clinical trials began before this point (11.5 vs 8.5 years, p<0.0005). Technological maturation represents the longest stage of translation, and significantly impacts the efficiency of drug development.

## Introduction

There is little theoretical understanding why the many dramatic advances in biomedical science in recent decades have not produced proportional growth in the number of new therapies. The problem is well documented; the annual number of approvals of New Molecular Entities (NMEs) has been static [1], clinical development pipelines continue to have high attrition rates [2], and drug development costs are increasing exponentially [3]. While there has been extensive analysis of the clinical and regulatory stages of drug development [2, 4, 5], less is known about the basic and applied research stages of translational science that provide constructive insights into mechanisms of health and disease, identification of potential drug targets, or the initial discovery of promising lead compounds.

Evidence suggests that the inefficiency of these early stages may be limiting the pace of translational science. The FDA and others have noted the lack of growth in the number of drug approvals reflects an underlying paucity in the number of candidate products in the clinical pipeline. Similarly, the President's Council of Advisors on Science and Technology (PCAST) identified lack of validated targets as a major barrier to building robust clinical pipelines and developing new cures [6].

There has been little empirical or theoretical work on these early stages of translational science. Much of the existing literature is anecdotal, describing experiential insights into the obstacles and opportunities encountered by individuals or companies engaged in target validation, high-throughput screening, lead identification, or preclinical testing. Often, these analyses address the challenges of entrepreneurship and financing, rather than how scientific insights are translated into products [4].

Theories of innovation arising from other technology sectors suggests that the ability to design and develop successful products is often dependent on a high degree of technological maturity [7–9]. Innovation research shows that technologies classically mature through a technology growth cycle, which can be quantitatively modelled as an “S-curve”. This research shows that novel technologies arise from precursor studies through scientific insights or inventions, which initiate a period of exponential technical growth. As this new technology advances and becomes established, technological progress slows and approaches a limit. Beyond this point, technological progress comes predominantly through the initiation of new technologies which mature through a new cycle of growth. The critical observation is that, while nascent technologies embody the promise of superior product performance, early stage technologies commonly fail to generate products that can meet the performance or market standards set by more mature, established technologies [8, 9]. It is not until new technologies achieve a certain level of technological maturity that they consistently produce products that can meet, or redefine, these standards.

Bibliometrics uses the accumulation of knowledge in a defined field, measured by number of PubMed entries, as a metric for technological growth and maturation. Studies have shown that the accumulation of publications commonly exhibits a characteristic S-curve growth pattern similar to that seen for other technologies [10], and that this growth can be modeled as an exponentiated logistic function, which we have named the Technology Innovation Maturation Evaluation (TIME) model.

**Fig 1:**
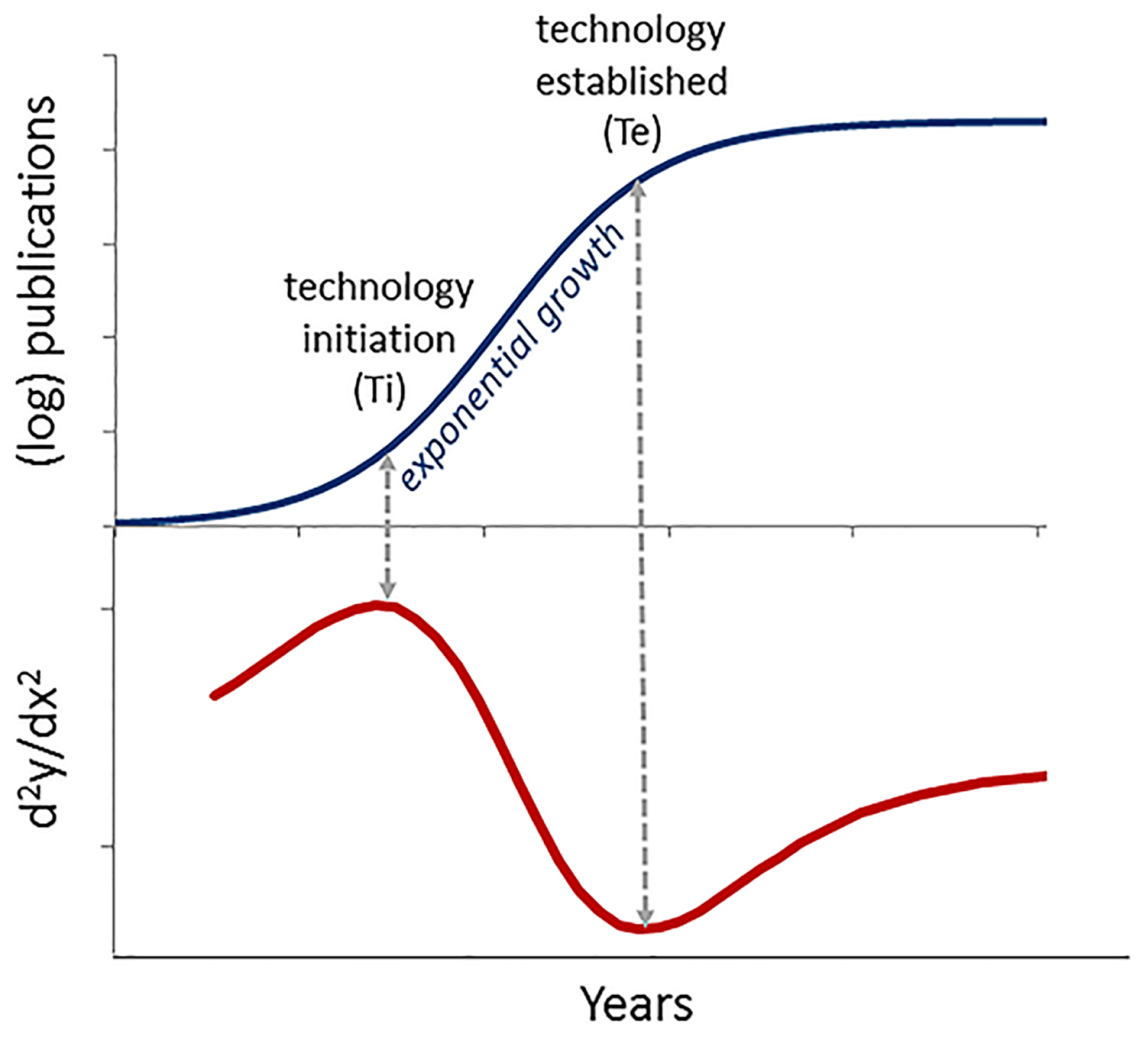
The TIME^tm^ model technology growth cycle. Top: Technology maturation is modeled as a best-fit, exponentiated logistic regression to the cumulative number of publications (N) over time. Bottom: The second derivative of this regression is used to identify the *initiation* point (point of greatest acceleration, d^2^log(N)/dx^2^ = max) and *established* point (point of maximal deceleration, d^2^log(N)/dx^2^ = min) for the technology. The *initiation* and *established* points bound a period of exponential growth, which subsequently slows as the technology approaches its limits.

This model provides statistically-defined metrics for both the point of *initiation* of new technologies and the point at which these technologies may be considered *established* (Fig 1). The *initiation* point is calculated as the date of maximum acceleration of publication activity, corresponding to the beginning of a period of exponential growth. The *established* point is calculated as the point where exponential growth ends, and there is maximum slowing of publication activity. The initial studies with this model showed that development programs involving monoclonal antibodies, nucleotide therapies, and gene therapies that were undertaken early in the growth cycles of these technologies uniformly failed, and that successful lead compounds entered clinical development only after the associated technology approached the *established* point [10, 11]. A similar pattern was observed for cancer therapeutics, where the many scientific and technological advances of the 1970s and 1980s generated few targeted or biological products until these technologies passed *established* point [12]. Significantly, no such association was seen for phenotypically discovered products, consistent with the fact that understanding of the targets or mechanism of action of phenotypic products is often unknown when the compound is discovered, and often follows from identification of bioactive compounds [13–16].

In the present work, we used this model to examine the timelines of translational science for the 138 new drugs and biological products approved by the FDA from 2010–2014. The goal of this work was to make an objective assessment of the time required for biomedical discoveries to be translated into successful biopharmaceutical products using state-of-the-art discovery and development technologies and within the contemporary regulatory environment. By doing so, we hoped to identify patterns of innovation in biopharmaceutical development as well as quantifiable milestones that might inform more efficient translational strategies, investments, and policies that could accelerate the translation of scientific discoveries for public benefit.

## Results

### Modeling the technology growth cycle

We identified 138 NMEs approved by the FDA from 2010 through 2014, excluding imaging agents, diagnostics, and reformulations or combinations of previously approved molecular entities. Of the 138 NMEs studied, 18 were approved in 2010, 25 in 2011, 37 in 2012, 20 in 2013 and 38 in 2014. This dataset of recently approved drugs best-reflects contemporary discovery and development practices and regulatory policies. The last decade has seen many innovations in regulatory science including safety science, adaptive clinical trial design and the use of biomarkers. Additionally, there has been widespread application of novel screening and research techniques, such as RNAi, genomics, rational drug design and microfluidic screening as well as novel chemical entities including the first bispecific antibody and antisense therapeutic. Finally, drug development has also been influenced by pressure from payers to demonstrate superior efficacy over existing drugs for pricing and reimbursement purposes [17, 18]. While the development and regulatory landscape continues to change, analysis of this most-recent period provides the best approximation of current practices.

Each NME was classified as “phenotypic”, “targeted”, or “biologic” based on the methods used for drug discovery or the molecular composition as described by Swinney [19]. Briefly, phenotypic products are discovered by screening for biological activities, without reference to a specific target or mechanism of action. In contrast, targeted drugs are discovered through *in vitro* assays involving specific targets or hypotheses regarding the molecular mechanism of action and require an understanding of the underlying biology in order to develop a molecule. Biologics include monoclonal antibodies (mAbs) as well as recombinant versions, or analogues, of natural bioactive proteins and are discovered by a process analogous to targeted screening. Using the classifications of drugs included in the work by Swinney [19], and applying their method to classify more recently approved drugs, provided an objective way to identify classes of drugs whose discovery was predicated on advancing knowledge of a drug target or disease mechanism, as well as those that might have been discovered independent of this knowledge [13–16]. Our hypothesis is that successful development of targeted and biological therapeutics would correlate with metrics of technological maturation, while no such relationship would be evident for phenotypic products. Of the 138 NMEs examined, 34 were classified as phenotypic, 64 classified as targeted, and 40 classified as biologics (S1 Table).

The technology associated with each NME was defined as the target used in screening assays for small molecule drugs or mAbs, the target of phenotypic NMEs (if known), or the natural counterpart of the protein comprising a non-mAb biologic. A unique target or natural biological entity was identified for 121 NMEs. For 17 phenotypic NMEs, the molecular target could not be unequivocally identified in the literature. PubMed searches were performed for each technology to quantify the accumulation of publications over time. The list of NMEs, associated target technologies and Boolean search terms is shown in S1 Table.

Technology growth curves were modeled as an exponentiated logistic regression (S-curve) using methods described previously. Target technologies associated with 102 products fit this model using goodness of fit measures (see Materials and methods). For each of these technologies, the *initiation* and *established* points bounding the exponential growth phase were determined (Fig 1, S1 Table)). Target technologies associated with 19 products did not exhibit a logistic growth pattern and were not further characterized. Sample curves are shown in S1 Fig.

**Fig 2:**
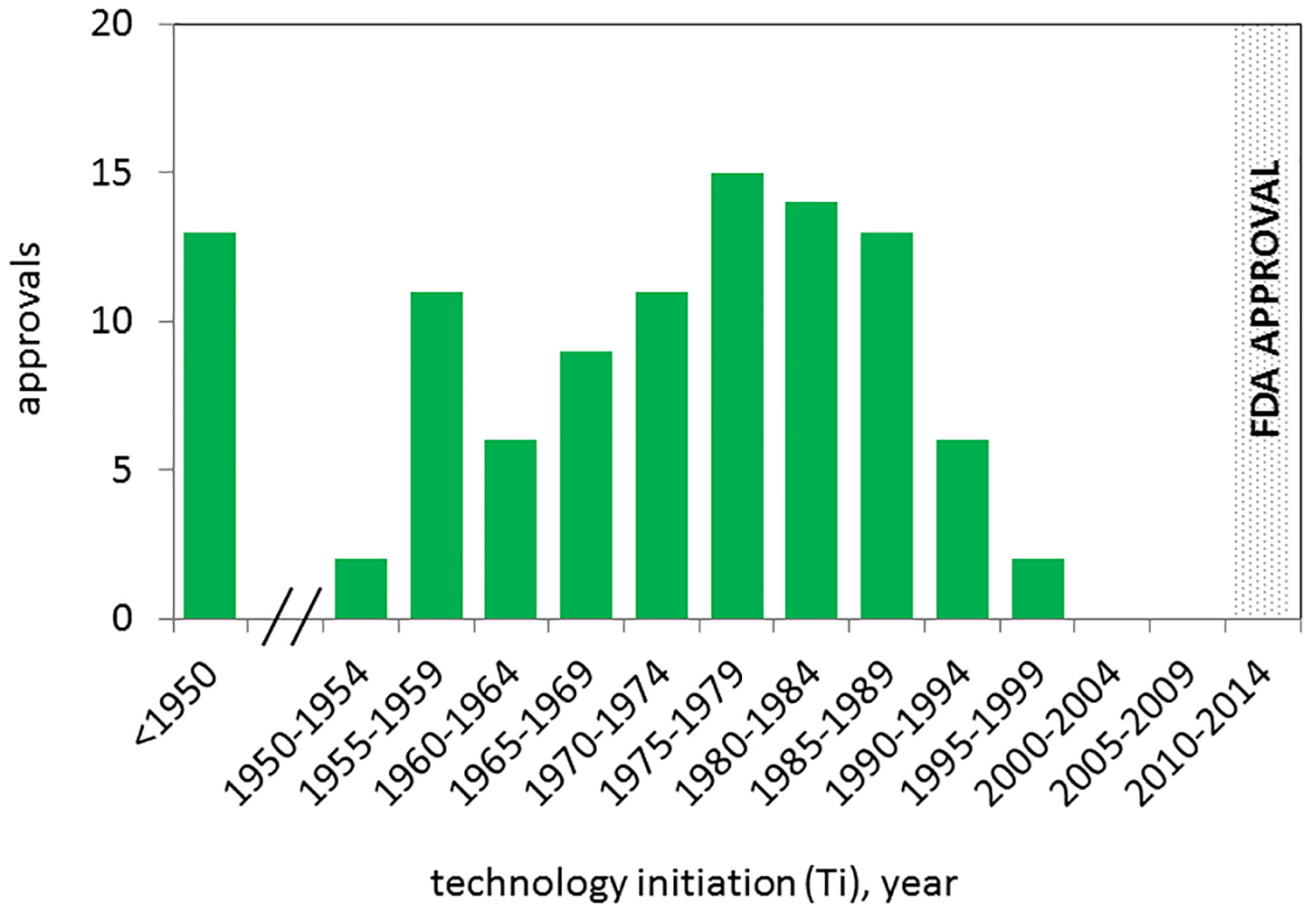
*Initation* (Ti) years for technologies associated with NME approvals 2010–2014. The distribution of technology *initiation* years for the drug target or biological entity associated with 102/138 NMEs approved by the FDA between 2010 and 2014.

The technology *initiation* points for targets associated with 102 NMEs are shown in Fig 2. The median *initiation* was in 1974. Of these, 91/102 NMEs were associated with targets that had an *initiation* point before 1985. For some NMEs, the *initiation* point corresponds with the publication date of works that described a specific target or gene product for the first time. For example, simeprevir (Olysio) and sofosbuvir (Sovaldi) are targeted to the hepatitis C virus polyprotein, which had a statistically defined *initiation* point of 1986. This date corresponds with the period between reports describing purification of the virus associated with “non-A non-B” hepatitis in 1985 [20] and the cloning of the viral genome in 1989 [21]. For others, the *initiation* point corresponds to the date of scientific advances that enabled target discovery. For example, ivacaftor (Kalydeco) is targeted to the Cystic Fibrosis Transmembrane Conductance (CFTR) protein (a chloride channel), discovered in the mid-1980s through genetic linkage studies and studies of ion transport. The statistically defined *initiation* point for CFTR was 1976, which corresponds in time with early genetic linkage studies using classical genetic markers [22]. Thus, the calculated *initiation* point recognizes that the technology growth cycle for CFTR did not begin with the isolation of this gene. Rather, the technology growth cycle was initiated by earlier research on biological processes that are associated with this protein, and the association of this locus with Cystic Fibrosis, and was already advancing exponentially when CFTR, itself, was identified. Considering non-genome based research, icabitant (Firazyr) is a bradykinin receptor B2 antagonist, used to treat acute attacks of hereditary angioedema. The *initiation* point for research on this target was 1980, which corresponds to early publications describing binding of bradykinin to the B2 receptor [23]. Similarly, tesamorelin (Egrifta) is a synthetic growth hormone releasing hormone, approved for reduction of excess belly fat in HIV infected patients. The *initiation* point for this biological product was 1967, which corresponds to publications describing the purification of GHRH from pigs and its biological effects in animal models [24].

### The timeline from initiation to drug approval

**Fig 3:**
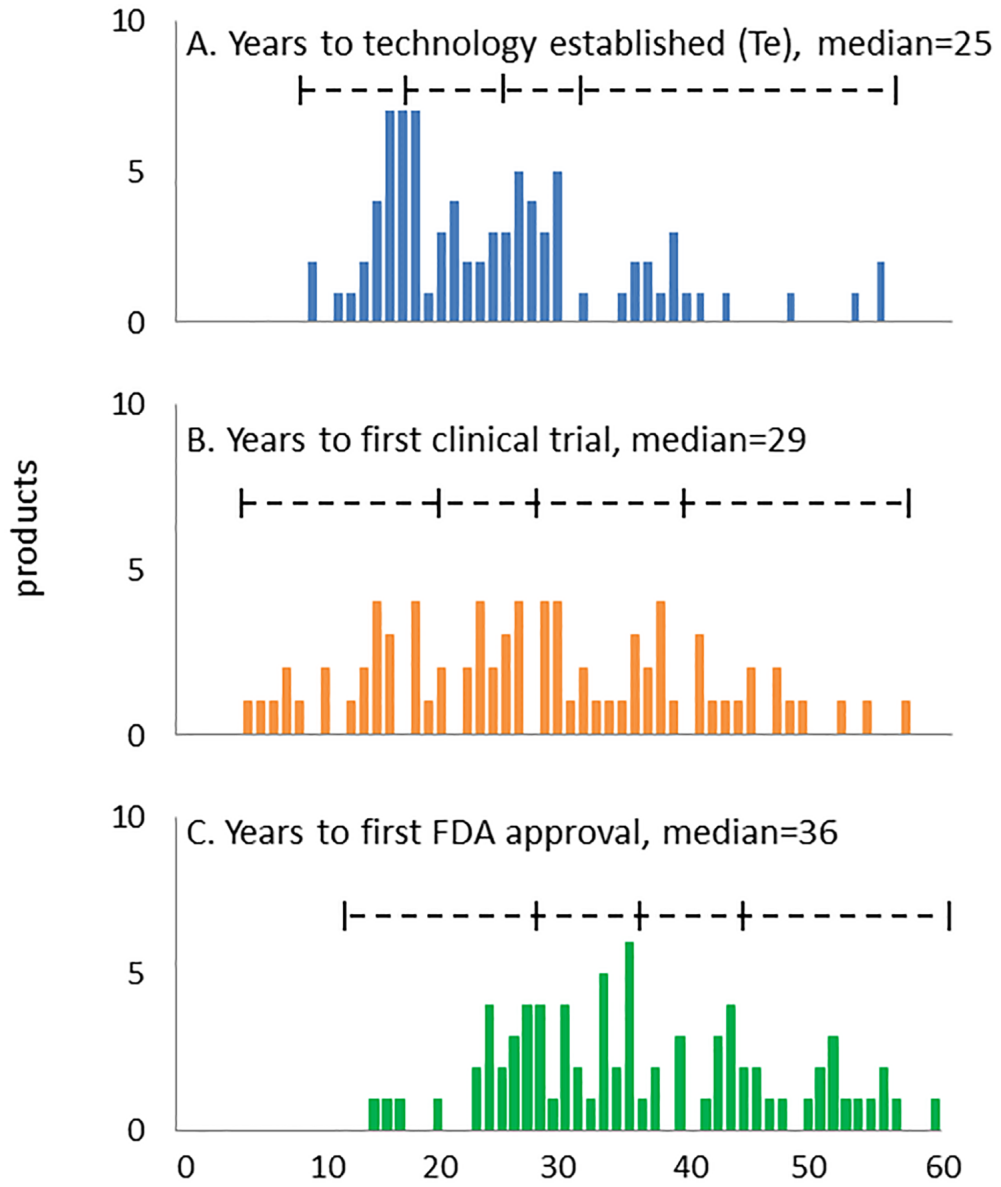
Time interval between technology *initiation* and metrics of translational science. (**A**) Time interval between technology *initiation* (Ti) and *establishe*d (Te) years. (**B**) Time interval between technology *initiation* and start of clinical trials. (**C**) Time interval between technology *initiation* and first drug approval. The median and quartile boundaries are shown for all technologies with *initiation* after 1950.

Fig 3 shows the timelines for translational science calculated from the *initiation* point. The median interval from the *initiation to established* points was 25 years (Fig 2A), the median interval from *initiation* to the start of clinical trials was 29 years (Fig 2B), and the median time from initiation to first approval was 36 years (Fig 2C). We did not observe any significant differences in these timelines for small molecules compared to biologics. The median time between the start of clinical trials and first approval for the NMEs in this study was 8 years (range 4 to 20, average = 9.4). These clinical development timelines are in line with previous reports [2].

Other studies have characterized the timeline of translational science starting with the publication dates of generally recognized seminal papers. Cockburn and Henderson examined a set of 21 drugs with the high therapeutic impact approved between 1965 and 1992 and reported that the average time from the “key enabling discovery” (i.e. fist report describing activity in a screen for phenotypic drugs or first description of a mechanism for “mechanism based,” targeted drugs) to market introduction was 25 years [25]. More recently, Eder et al. examined the interval from selected publications that described target or lead identification, to FDA approval for first in class drugs from 1999–2013 and reported a median interval of 22 years [26]. Our data set includes 40 of the first-in-class products studied by Eder as well as 10 follow-on products to those included in that study. There is a high degree of correlation between the statistically defined *initiation* point and the first publication empirically identified by Eder (t-test, p<1x10^-5^), though there was a 12 year average difference in these dates (t-test with hypothesized mean difference = 12 years, p< t-test, p<10^−12^) (data not shown). This interval is consistent with the observation that the statistically-defined *initiation* point corresponds to scientific discoveries or inventions that may precede, and enable, subsequent identification of specific targets or lead compounds. Further research is required to more accurately map specific, technical milestones to the metrics of the technology growth cycle.

### Impact of technological maturity on development

For 72/102, the start of clinical trials occurred after this *established* point (Table 1). We observed a similar ratio for targeted NCEs, phenotypic NMEs, or biologics. This ratio was also similar for first-in-class versus follow-on products (Table 1), consistent with the observation that most recent follow-on products have overlapping clinical development timelines with the first in class drug [27].

**Table 1:** Number of new chemical entities approved by the FDA 2010–2014 and average years in clinical trials when the first clinical trials were performed before the target technology was established (before Te) or after the technology was established (after Te).

Type	all	before Te	after Te	all	before Te	after Te	*p*
	# of compounds (2010–2014)			# years in clinical trials (average)			
All*	102	29	73	9.4	11.5	8.5	*0*.*0003*
Pheontypic	15	5	10	10.7	13.0	9.5	*0*.*2*^*NS*^
First-in-class	53	18	35	9.8*	9.5	8.7	*0*.*003*
Follow-on	49	12	37	8.9*	11	8.3	*0*.*01*
Targeted (NCE)+Biologic	87	24	63	9.4	11.5	8.5	*0*.*00004*
Targeted (NCE)	54	13	41	8.4	10.2	7.8	*0*.*01*
Biologic	33	11	22	10.2	12.4	9.3	*0*.*003*

Data are shown for 102 of 138 compounds approved by the FDA between 2010 and 2014. Of the remaining compounds, 17 are phenotypic compounds with an uncertain target/mechanism of action, and 19 exhibited a growth pattern that did not fit the exponentiated logistic model. *p* values from t-test.

*clinical timelines for first in class drugs compared to follow-on were not significantly different (t-test, p-value = 0.23)

NS = not significant

The length of time between the start of clinical trials and first approval was significantly shorter for products that entered clinical trials after the *established* point, than for those that entered development earlier (8.5 vs. 11.5, t-test, p<0.0005). The average difference was similar for targeted NCEs, phenotypic NCEs, and biologicals (approximately 3 years), but was not statistically significant for the small number of phenotypic NCEs (Table 1).

We examined whether there were significant differences in the translational timelines between first in class drugs and follow-on drugs approved from 2010–2014. As expected, the follow-on drugs approved in this interval were associated with targets that had earlier *initiation* points (median Ti first in class = 1978, follow-on = 1968), though there was no significant difference between the median Te for first in class drugs (2000) and follow-on drugs (1999). The average time between the *established* point and initiation of clinical trials was longer for follow-on drugs, but the difference was not significant (first in class = 4.1 yrs, follow-on = 5.5yrs, p = 0.37 (Table 1)). We would note that the time-delaminated study design means that the follow-on products do not correspond to first in class products in this study.

## Discussion

Despite dramatic advances in biomedical science and concerted efforts to streamline the clinical and regulatory stages of drug development, the efficiency of clinical development remains unchanged, and may actually be decreasing [1, 3–5]. At the same time, the cost of drug development continues to rise, with the most recent estimates average out-of-pocket cost for each new compound reaching $1.4 billion and fully capitalized costs reaching $2.6 billion [28]. There is accumulating evidence that there are barriers to development occurring in basic and applied research, which generates novel targets, target validation, and enables the subsequent stages of drug discovery and development. The PCAST report identified “rate-limiting knowledge gaps” that limited the number of compounds entering clinical development [4], while an Institute of Medicine report identified a “translational block” in the “transfer of new understandings of disease mechanisms gained in the laboratory” to testing in humans [5]. The present work was undertaken to better understand the relationship between these early stages of enabling research and the efficiency of subsequent development.

Our analysis is grounded in theories of innovation, which posit that new technologies mature through a characteristic growth cycle (S-curve), and that the ability to develop successful products from such technologies is related to the level of maturity. Previous studies have extended these principles to biopharmaceutical development [10, 11] using a bibliometric-based analytical model for the maturation of biomedical technologies. The present analysis models the maturation of target-related technologies as a characteristic S-curve (exponentiated logistic) and identifies the *initiation* point where exponential growth in publications begins as well as an *established* point representing slowing of publication activity and end of exponential growth in publication activity.

This work explicitly examines the timelines of translational science for NMEs approved by the FDA between 2010 and 2014. We show that for the 138 NMEs approved in this interval, there were 102 distinct targets, of which 90 exhibited a growth pattern that could be modeled as an exponentiated logistic function with acceptable fit/error. Using this model, we observed a strong association between maturity of the target technology and timelines of translational science for 102/138 NMEs approved from 2010–2014. There are two aspects to this observation.

First, the majority (73/102) only entered clinical trials after the target technology was *established*, and none of the NMEs approved 2010–2014 were approved before this point. We do not know how many failures might be associated with the lack of maturity of the target technologies. We would caution that these data also do not establish a causal relationship between the maturity of target technologies and clinical failure. Certain targets are traditionally considered “undruggable” due to their biochemical or biophysical characteristics, and some technologies, such as ribozymes, never generate therapeutic products. Moreover, clinical leads may fail for many reasons unrelated to the target such as chemical instability, disadvantageous pharmacokinetics, or idiosyncratic toxicities, and up to one third of leads may fail for “commercial” reasons related to the sponsor’s strategy or finances [2]. For example, data has shown that the probability of success in phase 2 is significantly lower when the trials are sponsored by smaller, biotechnology companies with limited capital resources [29]. Further studies, with cohorts of failed compounds, are required to sort out the relative contribution of technological maturity to development success or failure.

The data generated from this model also do not suggest that technological maturity is sufficient for success, and there is wide variation in the timelines observed between the established point and first approval of NMEs associated with these technologies. A closer examination of the four products that were approved within five years of the *established* point indicates that two were phenotypic (dimethyl fumarate (Tecfidera) and fingolimod (Gilenya)). The other two were denosumab (Prolia), a monoclonal antibody targeted against RANKL, a member of the TNF family of proteins, and ruxolitinib (Jakafi), a small molecule targeted against JAK protein kinases. Both RANKL and JAK are members of large superfamlies of proteins that had been successfully targeted with other therapeutics. More research is required to fully understand how the maturity of the target technology, the maturation of technologies related to the chemical entity, and other information known to be associated drug development such as Lipinski’s “rule of 5” [30] and AstraZeneca’s “5 R’s” [31],collectively contribute to the probability of successful development.

Second, data from this model shows the timeline of clinical development averaged three years shorter for products that entered clinical trials after the *established* point than for those that entered clinical trials when the target technology was less mature. This result suggests that the efficiency of clinical development was greater as the target technology passed the *established* point. To put the significance of this three year difference in context, DiMasi has examined the economic benefit of improving the timelines for drug development [32]. Based on his calculations, and using the most recent estimates for the fully capitalized cost of drug development [28], the 3 year (27–35%) average difference in drug development timelines observed here represents a cost differential of $100–200 million dollars in development costs. Many different factors may impact development timelines, ranging from the therapeutic area [33], small molecules versus biologicals, and various tracks for FDA review including Orphan drug designation, Expedited Drug Development, Accelerated Approval, Fast-Track designation, Priority Review, and Breakthrough Designation. Our data shows no significant difference between NCEs and biological products or between first in class drugs compared to follow-on products, but further [19] research would be required to sort out the multiplex effects of these factors on development timelines.

Analytical modeling of technology maturation provides an objective means for measuring the timelines of translational science. While it is more common to measure translational progress by tangible measures such as the dates of seminal publications or phases of clinical investigation, such metrics are inherently biased by the differential transparency of research performed in academic, start-up, or large corporate environments, differing standards for progressing through phased clinical trials based on the size or business models of the sponsoring corporation, as well as the subjective nature of *a posteriori* assessments of the importance of selected papers and milestones in successful development programs. Moreover, analytical metrics that might be calculated in real-time through the translational process have the potential to provide strategic guidance for development decisions and planning. While we have not explored the predictability of the current analytical model, the present results suggest that suitable metrics of technological maturity could be useful in handicapping the likelihood of clinical success and optimizing the timeline of clinical development.

This work focuses specifically on the maturation of technologies related to the drug target or, in the case of biologics, the natural counterpart of the protein. It is likely, however, that a complete picture of drug development requires a multifactorial analysis of technology related to the target, target families chemical entity, disease association, and ancillary technologies for production, delivery, and formulation. For example, we have previously examined the maturation of technologies related to monoclonal antibodies, gene therapies, and nucleotide therapeutics since the 1970s, showing that these technologies were successfully developed only after achieving a requisite level of maturity [10].

In the context of this dataset and analytical model, it is of interest that there a clear association between the maturation of technology associated with the target and the efficiency of drug development. We might speculate that this strong association with maturation of target-related technology reflects the established state of many complementary technologies such as recombinant protein production, monoclonal antibodies and small molecules, many of which are based on long-established chemistries, for example the “rule of 5” [30] We believe a multifactorial analysis of the different technologies that contribute to a drug would provide a more accurate description of the timelines of translational science [2].

The central observation in this work, namely the relationship between target technological maturity and effective biopharmaceutical development, is not surprising. There is a colloquial understanding that many clinical trials fail because the biology is “too complex” or “unpredictable”. While we do not yet fully understand the dynamics underlying the “S-curve” pattern of publications, we can postulate that the rate of publication continues to increase as long as new research questions continue to emerge, and that publication activity begins to slow as more of these questions are answered. Thus, the maximum slowing of publication activity at the *established* point could reflect the point at which the biology is no longer “too complex,” and quality of target validation, lead identification, and clinical trial design improves significantly. These data suggest that this is equally true for biological products as for small molecules.

This work emphasizes the importance of considering the complete timeline of translational science from the initial insights or inventions that give rise to a new technology, through to the launch of new products based on these technologies. The observation that the median time from target technology *initiation* to first clinical entry was 3–4 times longer than the timeline of clinical development, suggests that initiatives aimed at strategies for accelerating technology maturation could have a proportionally greater effect on the rate of translational science than those aimed exclusively at clinical development. Thus, research aimed at understanding the dynamic nature of technology maturation and its relationship to successful product development should be a high priority. The present observations point to the critical importance of consistent funding for nascent stage technologies to ensure their continued, unimpeded maturation, and also to the need for closer alignment between the basic and applied science that contributes to the maturity of technologies, and the strategic needs of product development.

## Materials and methods

### Data sources

NMEs approved by the FDA between 2010 and 2014 were identified from FDA.gov. Timelines of clinical development from the first clinical entry for any indication, to FDA approval were identified in PharmaProjects. To classify the NMEs as targeted, phenotypic, or biologic we used the method described by Swinney [19]. Briefly, this method involves a literature review to identify papers describing discovery of the lead compound. If the compound was discovered using a targeted screen of a chemical or MAb library, or is a biological copy or analogue of a naturally occurring biological product, the NME is classified as targeted.” If the compound was discovered based on a biological activity in vivo or in cell culture, it is classified as “phenotypic.”

For each monoclonal antibody, targeted and phenotypic NME, the associated technology was the drug target as identified in PharmaProjects or by a review of the literature. For biologics other than monoclonal antibodies, the associated technology was the biologic entity comprising, or analogous to, the product. Boolean search terms (S1 Table) were developed for each technology and the annual number of publications in PubMed was determined. Numerical modeling is performed from the first year of continuous publications identified in the literature, and excludes earlier publications separated by years with no publication activity. The discontinuous data points are excluded to minimize bias associated with incomplete ascertainment of relevant papers in earlier years due to immature vocabularies and/or the absence of abstracts. We have found that the early publication record is often very messy, and have chosen a rule-based approach to minimize this bias.

### Analytical modelling

An exponentiated logistic function was used to model publication growth:

$$N=L^{\left(\frac{1}{1+e^{-r\left(t-t_{0}\right)}}\right)}$$

Which also has the form:

$$logN=\frac{\text{log}L}{1+e^{-r\left(t-t_{0}\right)}}$$

Where *N* is the number of publications, *L* is the presumed upper limit of publications, *r* is the growth rate, *t* is time, and *t*_0_ is midpoint of exponential growth. The parameters were fit to time series publication data using a non-linear least squares implementation of the Levenberg-Marquardt algorithm, which can be found at: http://lmfit.github.io/lmfit-py/

This asymmetric sigmoidal function, exhibits the common logistic sigmoid function over log scales. This gives it property of having a symmetric growth phase that is exponential on average. The *initiation* and *established* points, representing the beginning and end of exponential growth or log*N*”(*t*)_max,min_ (Fig 1) can be analytically determined by:

$$Established,Initiation=t_{0}\pm \frac{\text{acosh}\left(2\right)}{r}$$

Standard errors from the Levenberg-Marquardt analysis were used to educate a Monte-Carlo simulation of the fitted parameters. The average standard error of this simulation was used as metric for goodness of fit. Generally, curve fits were considered to be valid if this error metric was less than 10%.

## Supporting information

S1 Table List of NMEs approved from 2010–2014, associated target technologies, search terms, molecular classification,technology timelines (Ti and Te) and clinical development timeline. For this analysis, we have explored many different equations that might be used to model technology growth, including functions fit to the cumulative number of publications (exponential, logistic, Richardson, Gompertz, exponentiated logistic, and exponentiated Gompertz). Both the exponetiated Gompertz and exponentiated logistic functions fit the majority of datasets examined. Both are routinely calculated in our analysis. We show the Ti, Te and residual mean square error for both functions. For the technologies in this study, the average residual mean square error was marginally better with the exponentiated Gompertz function (199) than EL (226). Our preference for the exponentiated logistic function over the exponentiated Gompertz function is based on two “ground truth” experiments that are routinely performed when testing the applicability of the model. First, we search the literature for historical review articles, and ask whether the estimated *initiation* point corresponds to seminal research advances highlighted by experts in the field. We find that the estimate of *initiation* provided by the exponentiated logistic model most often corresponds to seminal advances highlighted in review articles. Second, based on the timelines described in review articles and the titles/abstracts of articles identified in the PubMed search, we ask whether significant advances were made before the estimated *initiation* point. Across all technologies studied, we find that the estimates of *initiation* provided by the exponentiated Gompertz function often post-date an extended period of rapid advance in the field.
(XLSX)

S1 Fig Examples of technology growth curves. Markers show cumulative publication data for a given technology from Pubmed. Solid line shows model of publication growth using an exponentiated logistic function (see Methods for formula). Note that data are shown on a log scale, so that residuals at the low end of the model (e.g. 10^1^−10^2^ papers) appear disproportionately large in this representation, but actually have smaller residuals, because of the very small numbers of papers, relative to the residuals at the high end when there are 10^3^−10^5^ papers.
(TIF)
