# Supplementary figures and images for: Timelines of translational science: From technology initiation to FDA approval
Source: PLoS One. 2017 May 8;12(5):e0177371. doi: 10.1371/journal.pone.0177371 (PMC5421779; doi:10.1371/journal.pone.0177371)

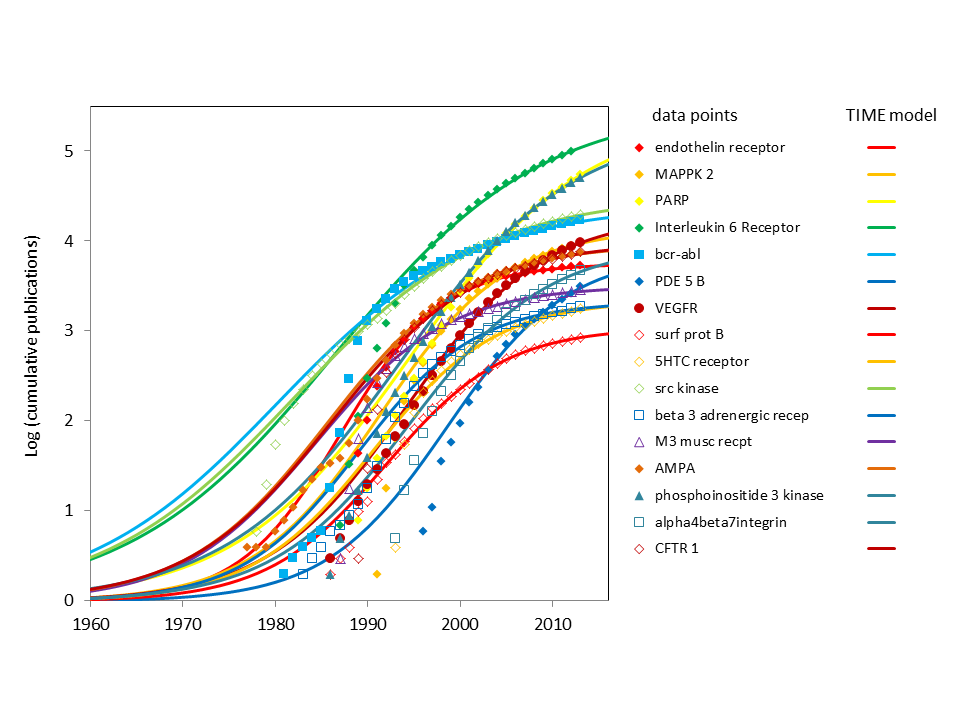

Supplement: S1 Fig — Markers show cumulative publication data for a given technology from Pubmed. Solid line shows model of publication growth using an exponentiated logistic function (see Methods for formula). Note that data are shown on a log scale, so that residuals at the low end of the model (e.g. 101−102 papers) appear disproportionately large in this representation, but actually have smaller residuals, because of the very small numbers of papers, relative to the residuals at the high end when there are 103−105 papers. (TIF) [file pone.0177371.s002.tif]
